# Increased Intraepidermal Nerve Fiber Degeneration and Impaired Regeneration Relate to Symptoms and Deficits in Parkinson's Disease

**DOI:** 10.3389/fneur.2019.00111

**Published:** 2019-02-14

**Authors:** Maria Jeziorska, Andrew Atkinson, Lewis Kass-Iliyya, Saad Javed, Christopher Kobylecki, David Gosal, Andrew Marshall, Monty Silverdale, Rayaz A. Malik

**Affiliations:** ^1^Division of Cardiovascular Sciences, University of Manchester, Manchester, United Kingdom; ^2^Department of Neurology, Greater Manchester Neuroscience Centre, Salford Royal NHS Foundation Trust, Salford, United Kingdom; ^3^Division of Neuroscience and Experimental Psychology, Manchester Academic Health Science Centre, University of Manchester, Manchester, United Kingdom; ^4^Department of Clinical Neurophysiology, Central Manchester NHS Foundation Trust, Manchester, United Kingdom; ^5^Weill Cornell Medicine-Qatar, Doha, Qatar

**Keywords:** Parkinson's disease, skin biopsy, small fiber neuropathy, intraepidermal nerve, motor, autonomic

## Abstract

**Background:** Previous studies have shown cutaneous small fiber pathology in patients with Parkinson's disease (PD). These studies have focused on nerve degeneration, but recent reports suggest that nerve regeneration may also be important in PD pathology.

**Objective:** To establish the extent of intraepidermal nerve fiber (IENF) degeneration and regeneration and its relationship to clinical and neurological deficits in Parkinson's disease (PD).

**Methods:** Twenty-three PD patients and 10 age-matched controls underwent skin biopsy and assessment of somatic and autonomic symptoms and deficits. We have assessed Intraepidermal Nerve Fiber Density (IENFD) using standard PGP9.5 staining and GAP-43 to assess Mean Axonal Length (MAL) and Intraepidermal Total Nerve Fiber Length (IETNFL).

**Results:** IENFD (*p* < 0.0001), MAL (*p* < 0.0001), IETNFL/Area (*p* = 0.009), and IETNFL/Length (*p* = 0.04) were significantly reduced in patients with PD compared to controls. IENFD correlated significantly with disease duration (*p* = 0.03), cumulative levodopa dose (*p* = 0.02), Unified Parkinson's Disease Rating Scale, Part III (UPDRS-III) (*p* = 0.01), Schwab and England Activities of Daily Living (ADL) (*p* = 0.03), NSP (*p* = 0.03), and 30:15 ratio (*p* = 0.03). IETNFL/Area correlated with the Autonomic Scale for Outcomes in Parkinson's Disease (SCOPA-AUT) (*p* = 0.03) and Diabetic Neuropathy Symptom score (DNS) (*p* = 0.04) and IETNFL/Length correlated with DNS (*p* = 0.03). MAL correlated with SCOPA-AUT (*p* = 0.01), DNS (*p* = 0.02), and DB-HRV (*p* = 0.02).

**Conclusion:** Increased IENF degeneration and impaired regeneration correlates with somatic and autonomic symptoms and deficits in patients with PD.

## Introduction

Parkinson's disease (PD) affects not only the central, but also the peripheral nervous system (1). Indeed, peripheral neuropathy (PN) has been reported in 55% of patients with idiopathic PD (2) and a recent systematic review has shown that 56.9% have biopsy proven small fiber neuropathy (3, 4). Pilomotor, sudomotor, and vasomotor dysfunction occur in patients with early PD (5). A recent study has shown a reduction in intraepidermal nerve fiber density (IENFD) and a loss of autonomic nerves to blood vessels, sweat glands, and arrector pili muscles and Meissner corpuscles and their myelinated endings in patients with PD compared to controls. Furthermore, the study also showed reduced IENFD on the predominantly affected and unaffected sides (6).

Over the last 4 years several studies have co-localized α-synuclein to nerves in skin biopsies (7, 8). However, deposition of phosphorylated and unphosphorylated α-synuclein was not associated with dermal autonomic denervation (3, 9) duration or severity of PD (10) or the side with the greatest motor deficits (11). Wang et al. have reported that α-synuclein deposition is increased in cutaneous sympathetic but not in sensory nerve fibers (3). Indeed, the utility of assessing peripheral α-synuclein deposition has been questioned, with a call for large multicentre trials using stringent detection and assessment methodology (12).

IENFD is the gold standard measure for small fiber neuropathy (SFN) (13) and has been advocated to study patients with PD (1, 7). Indeed, a recent study has shown that 82.1% of patients had reduced IENFD, whilst only 64.3% had reduced contact heat-evoked potentials and 32.1% had abnormal thermal thresholds (14). In our recent studies in patients with PD we have demonstrated a reduction in IENFD and corneal innervation, which was related to autonomic dysfunction (15) and perception of affective touch (16).

Traditionally, the morphological quantification of IENF has relied on immunostaining nerves with the pan-axonal marker, Protein Gene Product 9.5 (PGP 9.5) to derive a density of nerve fibers crossing the basement membrane into the epidermis as a marker of nerve degeneration. No previous studies have quantified detailed intraepidermal nerve fiber morphology, such as mean axonal length (MAL) or Intraepidermal Total Nerve Fiber Length (IETNFL) in PD.

Furthermore, recent studies have suggested that in early PD nerve regeneration may compensate for nerve degeneration and this regenerative capacity may decline over time (15, 17). No previous studies have directly assessed nerve regeneration in PD. Growth-associated protein-43 (GAP-43) was first described in growing nerve cones and has been shown to play a crucial role in neuromodulation and axonal regeneration in both animal and human studies (18, 19). GAP-43 staining has been applied to show altered cutaneous nerve fiber regeneration in patients with painful diabetic neuropathy (20). We have recently immunostained with GAP-43 and quantified Intraepidermal Total Nerve Fiber Length (IETNFL) and demonstrated increased epidermal nerve regeneration in patients with sarcoid neuropathy after treatment with Cibinetide (21).

In the present study we have undertaken detailed quantification of both intraepidermal nerve fiber degeneration and regeneration after immunostaining with PGP 9.5 (IENFD) and GAP-43 (MAL, IETNF). We have also related these markers of degeneration and regeneration to clinical and neurological deficits in patients with PD.

## Methods

### Standard Protocol Approvals, Registrations, and Patient Consents

The study was approved by NRES committee/North West (Ref. No 12/NW/0086). All participants gave their written informed consent.

### Study Population

Twenty-three patients (13 males, 10 females) fulfilling the UK Brain Bank criteria for the diagnosis of Parkinson's disease were recruited from neurology clinics after screening for other causes of peripheral neuropathy (cancer, chemotherapy, diabetes, impaired glucose tolerance, alcoholism, vitamin B6 and B12 deficiencies and autoimmune conditions). Ten age-matched healthy volunteers, non-related to the PD patients, served as controls.

### Clinical and Neurological Evaluation

PD duration was calculated in years from the onset of symptoms to the date of assessment. Patients were assessed in the “ON” state. The Unified Parkinson's Disease Rating Scale, Part III (UPDRS-III) (22) was used to rate motor symptom severity. The levodopa dose was expressed as total dose from the date first prescribed till the date of assessment.

Non-motor symptoms and pain were assessed using the non-motor symptoms scale (NMSS) (23), King's Parkinson's Pain Scale (KPPS) (24), Neuropathy Symptom Profile (NSP) (25), Diabetic Neuropathy Symptom score (DNS) (26), Schwab and England activities of daily living (ADL) scale and short form of McGill Pain Questionnaire (SFMPQ) (27). The Autonomic Scale for Outcomes in Parkinson's Disease (SCOPA-AUT) (28) was used to assess autonomic symptoms. Parasympathetic dysfunction was assessed by measurement of deep breathing heart variability (DB-HRV) and the heart rate ratio of the R-R interval at the 30th and 15th beat response to standing (30:15 ratio), using an ANX 3.0 autonomic nervous system device (ANSAR Medical Technologies Inc., Philadelphia). Blood pressure and heart rate in the supine position and after standing for 5 min were measured to evaluate for postural hypotension.

All patients underwent assessment of the neuropathy disability score (NDS) (29), vibration perception threshold (VPT) using a Neurothesiometer (Howell, Scientific Laboratory Supplies, Nottingham, U.K.) on both feet; thermal perception thresholds (warm [WT] and cold [CT]), heat-induced pain (WIP) and cold-induced pain (CIP) on the dorsum of the foot using a MEDOC TSA II (Medoc Ltd., Ramat-Yishai 20095, Israel) and nerve conduction studies were performed in the lower limbs.

### Skin Biopsy

Patients underwent 3 mm skin punch biopsies from the dorsum of each foot 3 cm above the third metatarsal. The samples were immediately fixed in 4% paraformaldehyde, cryo-protected in graded solutions of sucrose, frozen and stored at −80°C. The biopsies were sectioned perpendicular to the epidermis at 50 μm thickness on a cryo-microtome. Six non-consecutive sections were immunostained with the pan-axonal marker PGP9.5 (Abcam, Cambridge, U.K.) and used for IENFD quantification according to international guidelines (13). Six non-consecutive sections were immunostained with GAP-43 (Novus Biologicals, Oxon, U.K.), a specific marker for new or recently regenerated nerve fibers (18). The nerve fibers were visualized using SG chromogen (Vector Laboratories, Peterborough, U.K.).

A pathologist blinded to patients' details performed analysis of the sections. When assessing the GAP-43 staining, special care was taken to orientate skin sections such that the surface of the epidermis was parallel to the upper limit of the photographed field. Sections were imaged using a Zeiss AxioImager M2 microscope (Carl Zeiss, Jena, Germany) equipped with a motorized stage and digital camera. Each image for analysis was formed from Z-stacked optical layers, so that each final image contained all nerve fibers present in the full depth of the 50 μm section (post-sample processing), and an average of 20 images was obtained from each biopsy. All image measurements were performed using the Zen 2 image analysis programme (Zeiss Microimaging, Jena, Germany). The total nerve fiber length (TNFL) in the epidermis was normalized per millimeter length (TNFL/Length) and mm^2^ area (TNFL/Area) of epidermis, according to our recently described method (21). The mean length of nerve fibers crossing the basement membrane (BM) into the epidermis (MAL) was measured on GAP-43 immunostained sections. All nerve fiber parameters were obtained as an average of measurements in skin biopsies for the left and right foot.

### Statistical Analysis

GraphPad Prism v. 7.0 (GraphPad Software, Inc., USA) was used to perform all statistical analyses. The Shapiro-Wilk Test was used to assess normality. Two-tailed unpaired *t*-test or Mann-Whitney rank test were used to compare control and PD nerve fiber measurements. *P*<*0.05* was considered to be significant. Cohen *d* was calculated to measure effect size. Two-tailed Spearman correlation was used to assess the relationship between nerve fiber measurements and clinical measurements. The two-stage step-up method of Benjamini, Krieger and Yekutieli for False Discovery Rate was used to correct for multiple correlation calculations. *q* ≤ *0.2* was considered to be significant.

## Results

Patients with PD had a median Hoehn and Yahr score of II (range I–III) and a mean UPDRS motor score of 25.96 ± 12.7, consistent with moderate disease severity (Table 1).

**Table 1:** Demographics, clinical characteristics and quantitative measures of neuropathy in patients with PD and control subjects.

	**Controls (*n* = 10)**	**PD patients (*n* = 23)**	***p*-value**
Gender	8 males, 2 females	13 males, 10 females	
Age	60.0 ± 6 (Range 51–70)	61.9 ± 7.8 (Range 49–77)	0.550
Disease duration (years)	–	6.4 (4.8)	–
UPDRS-III	–	25.96 ± 12.7	–
Hoehn and Yahr stage		I = 10, II = 9, III = 4	
Total cumulative levodopa dose (g)	–	685.2 (118.8)	–
SCOPA-AUT	–	15.87 ± 7.1	–
NMSS	–	61.83 ± 26.6	–
King's PD pain scale	–	23.05 ± 15.4	–
SFMPQ	–	23.0 ± 12.6	–
Neuropathy disability score	1 (1.8)	2 (4)	0.074
Neuropathy symptom profile	0 (0)	7.86 ± 4.9	<0.0001
Diabetic neuropathy symptom score	0 (0)	1 (3)	0.0049
DB-HRV (beats/min)	25.46 ± 11.7	15.6 ± 6	0.028
Vibration perception threshold (V)	9.43 ± 6.8	12.63 (9.8)	0.049
Supine/standing systolic BP (mm Hg)	134.2 + 18.0/139.8 + 26.9	129.4 ± 18.3/129.8 ± 21.98	0.4088/0.3223
Supine/standing diastolic BP (mm Hg)	72.8 ± 12.3/76.2 ± 12.8	75.8 ± 7.5/77.15 ± 11.8	0.4616/0.7537
Δ Systolic BP (supine-standing [mm Hg])	−5.6 ± 19.35	−1.4 ± 10.7	0.4406
Cold perception threshold (°C)	26.88 ± 2.5	24.8 (13.1)	0.020
Heat perception threshold (°C)	37.77 ± 2.5	41.54 ± 3.9	0.005
Cold pain threshold (°C)	9.79 ± 8.6	0 (5.6)	0.127
Heat pain threshold (°C)	46.57 ± 2.8	48.45 (3.7)	0.435
IENFD (no./mm)	9.98 ± 2.5	3.02 ± 1.6	<0.0001
IETNFL/area (μm/mm^2^)	13,236 ± 5,006	6,488 (9,518)	0.009
IETNFL/length (μm/mm)	858.5 ± 345.1	537.8 (508)	0.04
MAL (μm)	67.19 ± 12.2	32.24 (11.3)	<0.0001

*Mean ± SD shown for normally distributed data. Median (Interquartile range) shown for non-normally distributed data. Two-tailed unpaired t-test or Mann-Whitney rank test were used to compare means between control and PD measurements. P < 0.05 was considered to be significant. ADL, activities of daily living; BP, blood pressure; DB-HRV, deep breathing heart rate variability; DNS, diabetic neuropathy symptom; IENFD, intraepidermal nerve fiber density; MAL, mean axonal length; NMSS, Non-motor Symptoms Scale; PD, Parkinson's disease; SCOPA-AUT, scales for outcomes in Parkinson's disease-autonomic; SFMPQ, Short Form McGill Pain Questionnaire; IETNFL, total nerve fiber length; UPDRS-III, Unified Parkinson's Disease Rating Scale*.

### Neurological Symptoms, Deficits, Quantitative Sensory, and Autonomic Testing

The NMSS, King's PD pain scale; SFMPQ and NSP indicated painful symptoms. VPT (*p* = 0.05), CT (*p* = 0.02), and WT (*p* = 0.005) were significantly elevated in patients with PD compared to controls (Table 1). There was no significant difference in the systolic or diastolic blood pressure on standing or 30:15 ratio, but DB-HRV was significantly reduced (*p* = 0.03) in patients with PD compared to controls.

### Intraepidermal Nerve Fiber Morphology

**Figure 1:**
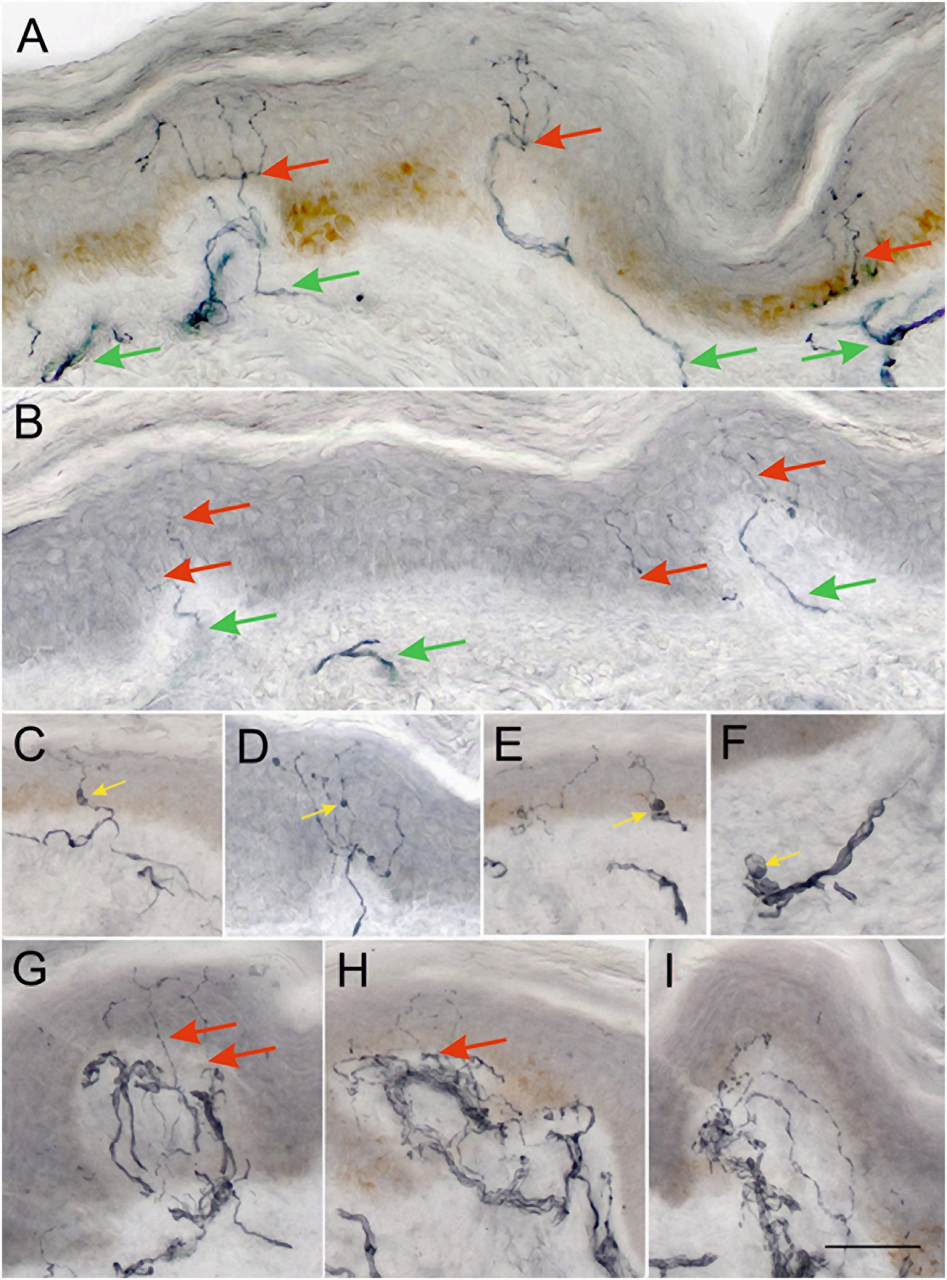
Representative examples of 50 μm sections from skin biopsies immunostained for GAP-43. Healthy control **(A)** shows numerous long branching intraepidermal nerve fibers (red arrows) reaching upper layers of epidermis and well-developed sub-epidermal nerve plexus (green arrows). Biopsy from a PD patient **(B)** showing scant, faintly staining intraepidermal nerve fibers (red arrows), and scant subepidermal plexus (green arrows). Images **(C–E)** show examples of axonal swellings along the IENFs and sub-epidermal nerve fibers **(F)** (yellow arrows). Images **(G–I)** show focal accumulations of nerve fibers in subepidermal area, which give rise only to occasional NFs crossing the BM into the epidermis (red arrows). Such areas are often separated by stretches of papillary dermis with very scant subepidermal plexus as in **(B)**. **(A–I)** were taken at the same magnification ×200, scale bar = 100 μm.

**Figure 2:**
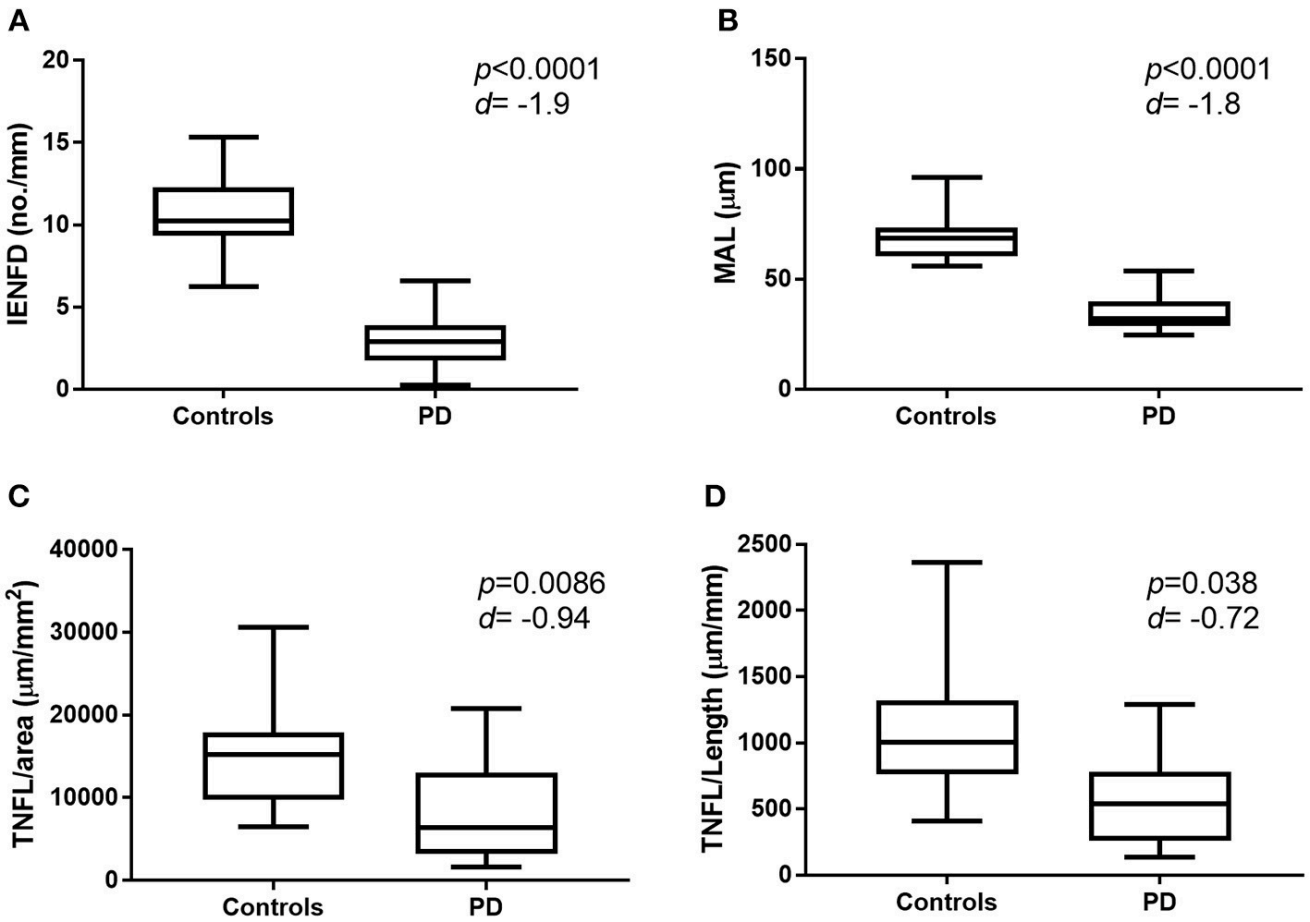
Graphic representation of nerve fiber measurement in healthy control subjects and patients with PD. Box and whisker plot showing mean, min and max values for IENFD **(A)**, MAL **(B)**, IETNFL/Area **(C)**, and IETNFL/Length **(D)**. Two-tailed unpaired Mann-Whitney rank test was used to compare means between control and PD nerve fiber measurements. *P*-value and effect size (*d*) shown (*d* > 0.5 = medium effect; over 1.0 = large effect). IENFD, intraepidermal nerve fiber density; MAL, mean axonal length; IETNFL, intraepidermal total nerve fiber length.

There was a significant reduction in IENFD (*p* < 0.0001), IETNFL/Area (*p* = 0.009), IETNFL/Length (*p* = 0.04), MAL (*p* < 0.0001) in patients with PD compared to control subjects (Table 1; Figures 1, 2).

#### Correlation of IENF Morphology With Clinical Data

IENFD correlated significantly with disease duration (*p* = 0.03), cumulative levodopa dose (*p* = 0.02), UPDRS-III (*p* = 0.01), Schwab and England ADL (*p* = 0.03), NSP (*p* = 0.03), and 30:15 ratio (*p* = 0.03). IETNFL/Area correlated with SCOPA-AUT (*p* = 0.03) and DNS (*p* = 0.04) and IETNFL/Length correlated with DNS (*p* = 0.03). MAL correlated with SCOPA-AUT (*p* = 0.01), DNS (*p* = 0.02) and DB-HRV (*p* = 0.02) (Table 2).

**Table 2:** Statistically significant Spearman's rank correlation between intraepidermal nerve fiber pathology, clinical and neurological deficits, quantitative sensory testing and autonomic deficits in PD patients.

		**Correlation coefficient (r)**	***p-value***	***q-value***
Disease duration	IENFD	−0.446	0.03	0.20
Levodopa dose	IENFD	−0.470	0.02	0.20
UPDRS-III	IENFD	−0.503	0.01	0.20
Schwab and England ADL scale	IENFD	0.457	0.03	0.20
NSP	IENFD	−0.480	0.03	0.20
30:15 ratio	IENFD	0.545	0.03	0.20
SCOPA-AUT	IETNFL/Area	−0.451	0.03	0.20
DNS score	IETNFL/Area	−0.460	0.04	0.23
DNS score	IETNFL/Length	−0.479	0.03	0.20
SCOPA-AUT	MDL	−0.525	0.01	0.20
DNS score	MDL	−0.512	0.02	0.20
DB-HRV	MDL	0.508	0.02	0.20

*Two-tailed Spearman rank correlation performed. Spearman rank correlation coefficient (r), p-value (p), and FDR q-value are shown. ADL, activities of daily living; DB-HRV, deep breathing heart rate variability; DNS, diabetic neuropathy symptom; IENFD, intraepidermal nerve fiber density; MDL, mean dendritic length; NMSS, Non-motor Symptoms Scale; PD, Parkinson's disease; SCOPA-AUT, scales for outcomes in Parkinson's disease-autonomic; SFMPQ, Short Form McGill Pain Questionnaire; IETNFL, intraepidermal total nerve fiber length; UPDRS-III, Unified Parkinson's Disease Rating Scale*.

## Discussion

This study demonstrates evidence of increased degeneration and impaired regeneration of IENF in patients with PD (4). The quantification of IENF was originally pioneered by Kennedy, who used PGP 9.5 staining to demonstrate loss of nociceptive and autonomic nerve fibers in the epidermal and subepidermal plexus (30, 31). Subsequently, IENF loss was standardized by manually counting IENF crossing the epidermo-dermal junction per length of epidermis (13) and global spatial sampling has been applied to estimate nerve length (32). In addition to demonstrating a reduction in IENFD in patients with PD, we now show a reduction in MAL, providing further evidence of IENF degeneration. MAL was originally introduced by Pittenger et al. (33) and termed mean dendritic length (MDL) and we have previously shown a significantly lower MDL in subjects with impaired glucose tolerance (34).

There is considerable debate regarding the utility of neuronal growth-associated protein, GAP-43 as a marker for nerve regeneration (18, 20, 32). However, it appears to be essential in regrowth of nerve fibers, be it post-traumatic or disease-related and also plays an important role in coordinating axonal outgrowth (18, 35). We have recently used GAP-43 staining in skin biopsies in patients with sarcoidosis related neuropathy and showed that GAP-43+ nerves increased in a dose dependent manner after a relatively short period of pharmacological intervention to stimulate nerve regeneration (21). Furthermore, Bonhof et al. have recently found a higher proportion of GAP-43+ compared to PGP9.5+ nerves in the skin of patients with diabetic neuropathy (20) and attributed this to differences in their respective rates of axonal transport.

In the present study, we show a reduction in both IETNFL/length and IETNFL/area, indicating impaired IENF regeneration in patients with moderate PD. Anand et al. have also used GAP-43 staining to show increased sensory or regenerating subepidermal nerve fibers in non-freezing cold injury (36).

The current data support our recent findings demonstrating an association between reduced IENFD and corneal innervation with autonomic dysfunction (15) and altered perception of affective touch (16) in patients with PD. We now extend these observations by showing that IETNFL and MAL are also related to autonomic deficits in PD patients. Small nerve fiber degeneration has been attributed to disruption in multiple metabolic and signaling pathways in the skin of patients with PD (37). Recent studies also show widespread small fiber pathology with a reduction in corneal nerve fiber density in patients with PD (38, 39). In a previous study we showed increased corneal nerve branch density and length, indicating increased small fiber regeneration, however, we could only show small fiber degeneration in the skin as we only evaluated IENFD (15). In the present study, we have used GAP-43 staining to assess small fiber regeneration in the skin and show a reduction in skin IETNFL, which indicates reduced intraepidermal nerve fiber regeneration.

We also demonstrate increased painful neuropathic symptoms, altered thermal thresholds and heart rate variability in addition to the typical motor deficits of PD and relate them to measures of IENF degeneration and regeneration. Surprisingly, IENFD in particular correlated with disease duration, cumulative levodopa dose and severity of motor disability as well as autonomic dysfunction, but not vibration or thermal thresholds. We have previously demonstrated significant associations between IENFD and motor deficits as well as severity of neuropathy and between corneal nerve loss with motor deficits and autonomic symptoms and deficits (15). However, we did not demonstrate any correlation between disease duration/motor severity and markers of small fiber regeneration. A longitudinal study is required to investigate changes in regeneration over time in PD.

A limitation of our study is the small number of PD patients who underwent skin biopsy, however this was comparable to previous studies. Nevertheless, we have expanded the morphological assessment of IENF pathology to demonstrate a reduction not only in IENFD, but also MAL, which represents a marker of more distal small fiber degeneration in patients with PD. In addition we have utilized GAP-43 to assess altered nerve fiber regeneration in patients with PD. These morphological measures of increased degeneration and impaired nerve regeneration correlate with clinical symptoms and deficits in PD.

## Conclusion

This is a small but detailed study, which shows that increased intraepidermal nerve fiber degeneration and reduced regeneration is associated with neurological deficits in patients with PD.

## Author Contributions

MJ analyzed skin biopsies. CK, DG, RM, and MS organized patient referrals and coordination of assessments. LK-I performed clinical assessments. SJ performed QST and AFT. AM performed the nerve conduction studies. AA performed statistical assessments. All authors contributed to drafting the manuscript and approved the final manuscript.

### Conflict of Interest Statement

MJ, AA, LK-I, DG, SJ, and AM declared that the research was conducted in the absence of any commercial or financial relationships that could be construed as a potential conflict of interest. CK received honoraria for educational meetings from UCB, Ipsen and Allergen; travel funding and advisory board from Britannia Pharmaceuticals. MS received honoraria for educational meetings from Medtronic and Bial. RM received honoraria for educational meetings from Pfizer and Novo Nordisk.
